# *Cronobacter sakazakii* and Microbiological Parameters in Dairy Formulas Associated With a Food Alert in Chile

**DOI:** 10.3389/fmicb.2018.01708

**Published:** 2018-07-31

**Authors:** Julio Parra-Flores, Fabiola Cerda-Leal, Alejandra Contreras, Nicole Valenzuela-Riffo, Alejandra Rodríguez, Juan Aguirre

**Affiliations:** ^1^Molecular Microbiology Laboratory, Faculty of Health and Food Sciences, Universidad del Bío-Bío, Chillán, Chile; ^2^Department of Agricultural Industry and Enology, Universidad de Chile, Santiago, Chile

**Keywords:** *Cronobacter sakazakii*, food alert, microbiological parameters, powdered infant formula, liquid dairy formula

## Abstract

The objective of this study was to evaluate the presence of *Cronobacter sakazakii* and microbiological parameters in dairy products associated with a food alert. Ninety dairy product samples were analyzed, including seven commercial brands and two product types (liquid and powdered) from four countries. Aerobic plate count (APC) and *Enterobacteriaceae* count were performed according to Chilean standards. *Cronobacter* spp. and *C. sakazakii* were identified by polymerase chain reaction real time amplification of *rpoB* and *cgcA* genes and the genotype by multilocus sequence typing. Eighty-eight percent of dairy products showed APC higher than the detection limit. Fifty percent of liquid commercial brand samples contained APC: 2.6, 2.3, 1.1, and 2.9 CFU/mL in brands A, C, E, and G, respectively. Results for powdered commercial brands were 3.0, 3.6, and 5.7 CFU/g in brands B, D, and F, respectively. Maximum count (5.7 CFU/g) occurred in brand F dairy product manufactured in Chile. *Enterobacteriaceae* were found in 55% of the samples, 64% in liquid and 51% in powdered commercial brands. In 50% of brands B, D, and E, samples contained 2.9, 2.8, and 2.7 log CFU/g, respectively. Only liquid commercial brands from the United States had *Enterobacteriaceae* values between 0.1 and 4.5 CFU/mL. Seventeen suspicious strains were isolated and nine were identified as *Enterobacter* spp. Only eight suspicious strains from four powdered commercial brands (Chile and Singapore) were confirmed as *C. sakazakii* by *rpoB* and *cgcA* gene amplification and *fusA* sequencing. *C. sakazakii* prevalence in the analyzed samples was 8.8%. There were 11% of powdered milk brands that contained APC between 4.0 and 4.7 log CFU/g and 55% of the samples contained *Enterobacteriaceae*. *C. sakazakii* was found in dairy products manufactured in Chile and Singapore. On the basis of this information, the Chilean Ministry of Health (RSA) decreed a national and international food alert and recalled all the product batches that resulted positive in the present study from supermarkets and pharmacies.

## Introduction

On June 2, 2017 the Chilean Ministry of Health issued a national and international food alert as a result of the presence of *Cronobacter sakazakii* in two powdered formula samples intended for children under 10. Researchers from the Universidad del Bío-Bío conducted a study which led to the food alert. This preventive measure was adopted because of the risk of disease associated with *Cronobacter* spp. and *C. sakazakii* in hypersensitive groups of the population (Food and Agriculture Organization and World Health Organization [FAO/WHO], 2008; Jason, 2015).

*Cronobacter* spp. was initially defined as the new bacterial species *Enterobacter sakazakii* by Farmer et al. (1980); it was later classified by Iversen et al. (2008) and Joseph et al. (2012a,b) as *Cronobacter* spp. and included seven species: *C. sakazakii, C. malonaticus, C. universalis, C. turicensis, C. muytjensii, C. dublinensis*, and *C. condimenti*.

*Cronobacter* spp. is considered as an emerging pathogen that is especially aggressive in hypersensitive individuals, such as children and the elderly (Fanning and Forsythe, 2008; Hunter and Bean, 2013). Newborns and the elderly are the population groups that are most affected by *C. sakazakii*, although the highest incidence and severity occurs in preterm infants (Hariri et al., 2013). Outbreaks have generally been the most common cause of infection (Jason, 2012). Clinical symptoms are mainly found in meningitis, septicemia, or necrotizing enteritis in infants (Block et al., 2002; Bowen and Braden, 2006), but diarrhea, urinary tract infection, and septicemia have also been observed. Mortality rates are associated with general infection (42–80%) and neonatal meningitis and septicemia (15–25%) (Holý and Forsythe, 2014).

The disease is associated with the consumption of rehydrated milk as a carrier of the pathogen, as well as the eventual involvement of utensils and equipment as reservoirs (Food and Agriculture Organization and World Health Organization [FAO/WHO], 2008; Kalyantanda et al., 2015). Since it is widespread, it can be isolated in powdered infant formula (PIF), rehydrated milk (R-PIF), infant cereals, various foods, water, surfaces, homes, and hospitals (Baumgartner et al., 2009). Even when the source of primary contamination is unclear (Norberg et al., 2012), some researchers suggest that the natural habitat is PIF manufacturing plants. This situation has been reported by Jacobs et al. (2011) and Li et al. (2014), who identified *Cronobacter* spp. in different parts of PIF plants in both China and Australia. It has also been strongly associated with by-products used in its formulation, which are also probable carriers (Jongenburger et al., 2011). The control of this pathogen in the first stages of PIF production is the most important step to reduce its incidence in the final product (Yan et al., 2012; Fei et al., 2015) because viable *Cronobacter* spp. strains have been found 2 years after the product was packaged (Caubilla-Barron and Forsythe, 2007).

Although updated detection and identification techniques are being used, there are still cases of disease and mortality every year (Norberg et al., 2012). It is therefore necessary to improve hygiene and the production process to reduce the impact of *C. sakazakii.* Biochemical tests (API 20E, RAPID, BIOLOG microarray), molecular confirmation of the *Cronobacter* spp. genus by polymerase chain reaction (PCR) (Lehner et al., 2004; Cetinkaya et al., 2012), and especially multilocus sequence typing (MLST) have been used to complement its identification. These techniques have allowed advances in correctly identifying it, and thus decreasing the possibility of false negatives (Baldwin et al., 2009; Joseph and Forsythe, 2011; Yan et al., 2015; Ogrodzki and Forsythe, 2017). Several primers have been generated to detect *Cronobacter* spp. by amplifying specific sequences of variable and conserved regions of 16S ribosomal rDNA of the bacterium (Lehner et al., 2004; Hassan et al., 2007), *OmpA* (Mohan-Nair and Venkitanarayanan, 2006), as well as others that are more specific, which detect *C. sakazakii* by *rpoB* (Stoop et al., 2009; Lehner et al., 2012; Li et al., 2016) and *cgcA* gene amplification (Carter et al., 2013).

Studies of *Cronobacter* spp. incidence in powdered milk have demonstrated a positivity range between 3 and 30% (Chap et al., 2009; Siqueira-Santos et al., 2013; Fei et al., 2017). Sáez et al. (2012) found 5% positivity of *Cronobacter* spp. in 80 PIF samples from a dairy processing plant in the Los Lagos Region in Chile. Parra-Flores et al. (2015a) found an incidence of 9.5% *C. sakazakii* in an exploratory study with a limited number of samples using MLST in PIF manufactured in Chile in 2014. If PIF samples manufactured in other countries are considered, incidence was 2.7% in all the analyzed PIF samples.

Given the need to ensure safety in PIFs, the FAO/WHO have held expert meetings to study cases of diseases related to its consumption, whether epidemiologically or microbiologically. Three categories of microorganisms were identified based on the soundness of evidence of a causal relationship between their presence in food and the disease: (A) microorganisms with clear causality evidence, enteric *Salmonella*, and *Cronobacter* spp. (*E. sakazakii*); (B) microorganisms in which causality is possible but has not yet been demonstrated, primarily from the *Enterobacteriaceae* family; and (C) microorganisms in which causality is less probable or has not yet been demonstrated, and have not been identified in PIF (Food and Agriculture Organization and World Health Organization [FAO/WHO], 2006; Jackson et al., 2015). The WHO therefore recommended the absence of *Cronobacter* spp., *Salmonella*, and *Enterobacteriaceae* in dairy products (Food and Agriculture Organization and World Health Organization [FAO/WHO], 2004, 2006).

Given that PIFs are not sterile foods, the determination of microbial indicators, such as aerobic plate count (APC) and *Enterobacteriaceae* (ENT), provides useful information about the hygienic conditions of their preparation or post-process contamination (Friedemann, 2009; Parra-Flores et al., 2015a; Heperkan et al., 2017).

*Cronobacter* spp. was not considered in the Chilean Food Sanitary Regulations (Reglamento Sanitario de los Alimentos, RSA) when the Chilean Ministry of Health decreed the food alert. The decision was taken due to the risk of disease associated with the pathogen described in the scientific literature (Jason, 2015), factors that affect PIF contamination (Parra-Flores et al., 2015b), and the variability in its cellular response (Parra-Flores et al., 2016). The PIFs associated with this alert are not only commercialized in Chile but throughout the Americas.

Therefore, the objective of this study was to evaluate the presence of *C. sakazakii* and the microbiological parameters of APC and ENT in dairy products associated with a food alert in Chile in June 2017.

## Materials and Methods

### Food Samples

Sampling was conducted from August 2016 to May 2017. Ninety samples were collected in four countries (United States, Singapore, Chile, and Holland), from three manufacturers (1, 2, and 3), seven commercial dairy brands (A, B, C, D, E, F, G) of which B, D, and F were powdered and A,C, E, and G were liquid products sold in supermarkets and pharmacies in Chile. All the analyses were performed in duplicate. The sampling criteria used as a reference were the standards of the Chilean RSA and CAC/RPC 66 of the Codex Alimentarius.

### Microbiological Quantification

The APC of the mesophilic microorganisms and ENT count were used. Quantification of both microbial groups and identification of isolated enterobacteria (including suspicious strains of *Salmonella* or *Escherichia coli*) were performed in the Accredited Food Testing and Certification Laboratory (LECYCA-UBB) and the Molecular Epidemiology and Microbiology Laboratory of the Universidad del Bío-Bío. References are NCh 2659 (2002) for AMC, NCh 2676 (2002) for ENT, NCh 2636 (2002) for *E. coli*, and NCh 2675 (2002) to isolate *Salmonella*.

### Isolation of *Cronobacter* spp.

The technique described by Parra-Flores et al. (2015a) was applied. For each sample, 225 mL of buffered peptone water (BPW) were added to 25 g of powdered infant formula (PIF) or dairy product (DP) and then homogenized in a stomacher at a mean velocity for 60 s. Liquid products in their original container were directly incubated at 37°C. Then 10 mL of each sample was inoculated after incubation at 37°C for 24 h in 90 mL *Enterobacteriaceae* enrichment broth (BD Difco, Sparks, MD, United States). A loop was extracted from the culture suspension and striated in Brilliance Chromogenic Agar CM 1035 (Oxoid Thermo Fisher, United Kingdom) at 37°C for 20 h. Five strains, presumed to be colonies of *Cronobacter* spp. (green or blue), were striated in trypticase soy agar (BD Difco, Sparks, MD, United States) to verify their purity prior to future analyses. The isolated strains were maintained in a strain collection and stored at -80°C.

### Identification of *Cronobacter* spp. and *Cronobacter sakazakii*

Genomic DNA of the suspicious strains was extracted and purified with the Ultra Clean^®^ Microbial DNA Isolation Kit (MO BIO Laboratories, Inc., Carlsbad, CA, United States). The strains were confirmed as *Cronobacter* spp. by *OmpA* gene amplification (Mohan-Nair and Venkitanarayanan, 2006) and later identified as *C. sakazakii* by qPCR amplification of *rpoB* and *cgcA* genes (Stoop et al., 2009; Carter et al., 2013) (**Table 1**) in the Stratagene Mx3000P qPCR System equipment (Agilent Technologies).

**Table 1:** Polymerase chain reaction (PCR) primers used in the study.

Identification	Gene	Primer sequence	Reference	Annealing temperature
*Cronobacter* spp.	*16S*	F: GCT YTG CTG ACG AGT GGC GGR: ATC TCT GCA GGA TTC TCT GG	Lehner et al., 2004	60°C
*Cronobacter* spp.	*OmpA*	F: GGATTTAACCGTGAACTTTTCCR: CGCCAGCGATGTTAGAAGA	Mohan-Nair and Venkitanarayanan, 2006	60°C
*Cronobacter sakazakii*	*rpoB*	F: ACG CCA AGC CTA TCT CCG CGR: ACG GTT GGC GTC ATC GTG	Stoop et al., 2009	67°C
*Cronobacter sakazakii*	*cgcA*	F: GTGGCSGGGTATGACAAAGACR: GGCGGACGAAGCCTCAGAGAGT	Carter et al., 2013	62°C
*Cronobacter sakazakii*	*fusA*	F: GAAACCGTATGGCGTCAG’R: AGAACCGAAGTGCAGACG	Baldwin et al., 2009.	58°C


### Sequencing of *fusA* Gene to Identify Species of *Cronobacter* spp.

The methodology described by Baldwin et al. (2009) was followed using PCR CORE Kit QIAGEN (Cat No. 201225) solutions. Amplified products were sent to MACROGEN in Korea for sequencing. Identification was performed with the free access online database https://pubmlst.org/cronobacter/ and BLASTn (NCBI).

### Bioinformatic and Statistical Analyses

The sequenced products were analyzed with the Gentle software and later aligned with the ClustalW software. A phylogenetic tree was constructed using the maximum likelihood method with the MEGA7 software. Statistical description included measures of central tendency, dispersion, and position for quantitative variables, while absolute frequencies and percentages were used for qualitative variables. The Mann–Whitney and Kruskal–Wallis tests were used for comparison purposes with the STATA 14 software at the significance level α = 0.05.

## Results

Of the 90 analyzed Chilean and foreign samples, 79 had APC. When analyzing APC for each DP commercial brand, no significant statistical differences were found (*p* > 0.05). However, half of the liquid DP commercial brands contained 2.6 log CFU/mL, 2.3 log CFU/mL, 1.1 log CFU/mL, and 2.9 log CFU/mL for brands A, C, E, and G, respectively. The powdered DP (including PIF) commercial brands had values of 3.0 log CFU/g, 3.6 log CFU/g, and 5.7 log CFU/g for brands B, D, and F, respectively. Positivity for ENT was found in all the evaluated brands. In the liquid DP brands A, C, E, and G, the sample means were 1.8, 0.8, <0.1, and <0.1 log UFC/mL, respectively. In half of powdered DP brands B, D, and E, samples contained 2.9, 2.8, and 2.7 log CFU/g, respectively. Statistical differences were only found in the ENT counts (*p* = 0.012). For DP manufacturers, company 2 had the highest count with 5.9 log CFU/g. *Enterobacteriaceae* counts were found in 55% of the total analyzed samples. Company 1 obtained the highest counts with 3.5 log CFU/g (**Table 2**).

**Table 2:** Aerobic plate count (APC) and *Enterobacteriaceae* (ENT) count for each commercial brand and manufacturer.

	Counts	
	APC log CFU/g Median (minimum–maximum)	ENT log CFU/g Median (minimum–maximum)
**Commercial brand**		
A	2.6 (1.7–5.7)	1.8 (1.6–4.5)
B	3.0 (2.3–4.1)	2.9 (1.0–3.6)
C	2.3 (2.0–5.8)	0.85 (0–2.9)
D	3.6 (1.3–5.9)	2.8 (1.0–4.4)
E	1.1 (0.9–5.8)	<0.1 (0.1–4.5)
F	5.7 (1.3–6.7)	2.7 (1.0–3.0)
G	2.9 (2.6–5.7)	<0.1
***p*-value^∗^**	ns	0.0121
**Manufacturer**		
1	3.0 (1.3–5.9)	2.6 (0–4.5)
2	5.7 (0.9–6.8)	2.7 (1.0–3.0)
3	2.9 (2.6–5.7)	<0.1
***p*-value^∗^**	ns	0.0107


*^∗^p-value according to Kruskal–Wallis test. Brands A, C, E, and G: liquid dairy products; Brands B, D, and F: powdered milk products; ns: non-significant*.

Regarding the country of origin (**Table 3**), Chile exhibited the highest APC count means with 5.0 log CFU/g and the United States showed the lowest count with 2.1 log CFU/g. The DP produced in Holland had the highest ENT counts followed by Singapore with 3.6 and 3.2 log CFU/g, respectively. The lowest count was obtained in the US with 0.1 log CFU/g. No significant differences existed in the APC and ENT counts for country of origin (*p* > 0.05). As for the type of DP, 100% of the liquid DP brands contained APC and 55% ENT counts. Only the liquid DP brands produced in the US had ENT counts with values between 0 and 4.5 log CFU/mL. The highest count in powdered DPs was obtained in Holland with 4.4 log CFU/g.

**Table 3:** Aerobic plate count (APC) and *Enterobacteriaceae* (ENT) count for each country.

	Counts	
	APC log CFU Median (minimum–maximum)	ENT log CFU Median (minimum–maximum)
**Country**		
Chile	5.0 (1.3–6.8)	2.7 (1.0–3.0)
Holland	3.6 (1.3–5.9)	2.8 (1.0–4.4)
Singapore	3.0 (2.3–4.1)	2.9 (1.0–3.6)
United States	2.1 (0.9–5.8)	1.7 (0–4.5)
***p*-value^∗^**	ns	ns


*^∗^p-value according to Kruskal–Wallis test; ^∗∗^p-value according to Mann–Whitney test. ns, non-significant.*

Of the total analyzed samples, 17 suspicious strains were isolated from the chromogenic agar. Nine were identified as *E. cloacae, Klebsiella pneumoniae, E. hormaechei*, and *Enterobacter* spp., whereas *Salmonella* spp. was not isolated in any of the samples. However, *E. coli* was identified in one powdered milk (PM) product manufactured in Chile.

Only eight suspicious strains from the PM from Chile and Singapore were confirmed as *Cronobacter* spp. by amplifying the *ompA* gene. These strains were subsequently confirmed as *C. sakazakii* through the amplification of the gene products for *rpoB* and *cgcA* by PCR in real time. One of the PM products in which *C. sakazakii* was isolated was intended for consumption by infants under 2 years (CH84), and another from Singapore was intended for consumption by children older than 1 year (CH65).

Furthermore, six more strains were confirmed, which were not part of the food alert because of their expiry date and were PM products manufactured in Chile (CH42, CH43, CH44, CH45, CH50, and CH85) (**Table 4**). Two more samples were also detected by real-time PCR with *Cronobacter* spp. from PIFs manufactured in Holland, but it was not possible to recover the pathogen from the samples.

**Table 4:** Identification of *Cronobacter* spp. and *Cronobacter sakazakii* by molecular amplification.

Strain	Gene amplification					Identification	
	16S	*OmpA*	*rpoB*	*cgcA*	*fusA* sequencing allelle	MLST database	BLAST
CH42	**+**	**+**	**+**	**+**	1	*Cronobacter sakazakii*	*Cronobacter sakazakii*
CH43	**+**	**+**	**+**	**+**	1	*Cronobacter sakazakii*	*Cronobacter sakazakii*
CH44	**+**	**+**	**+**	**+**	1	*Cronobacter sakazakii*	*Cronobacter sakazakii*
CH45	**+**	**+**	**+**	**+**	16	*Cronobacter sakazakii*	*Cronobacter sakazakii*
CH50	**+**	**+**	**+**	**+**	16	*Cronobacter sakazakii*	*Cronobacter sakazakii*
CH65	**+**	**+**	**+**	**+**	1	*Cronobacter sakazakii*	*Cronobacter sakazakii*
CH84	**+**	**+**	**+**	**+**	71	*Cronobacter* spp.	*Cronobacter sakazakii*
CH85	**+**	**+**	**+**	**+**	71	*Cronobacter* spp.	*Cronobacter sakazakii*


*Cronobacter sakazakii* incidence in the total evaluated samples was 8.8% (**Table 5**).

**Table 5:** Positivity of *Cronobacter sakazakii* for country of origin and product type.

Positivity of *Cronobacter sakazakii* in dairy product (DP) samples					
Country	*n*	Powdered infant formula	Liquid DP	Total	
		(+)	(+)	(+)	%
Chile	20	7	0	7	35
United States	25	NE	0	0	0
Holland	35	0	NE	0	0
Singapore	10	1	NE	1	10
Total	90	8	0	8	8.8


*NE, not evaluated.*

**FIGURE 1:**
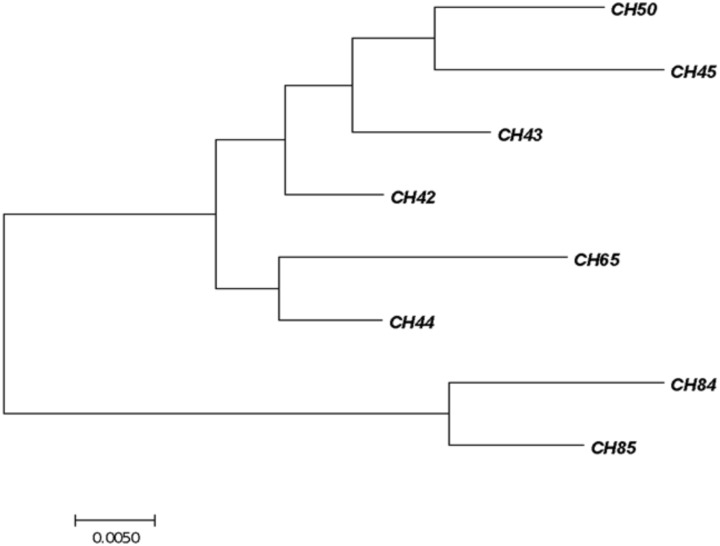
Phylogenetic tree of fusA sequencing identified as *Cronobacter sakazakii*. The tree with the highest log likelihood (–1450.2262) is shown. The initial tree for the heuristic search was obtained automatically by applying Neighbor-Join and BioNJ algorithms. The analysis involved 24 nucleotide sequences. There were 401 positions in the final dataset. Evolutionary analyses were conducted with the MEGA7 software program.

All the *C. sakazakii* strains were genotyped by sequencing the *fusA* gene using MLST in the database https://pubmlst.org/cronobacter/ and BLASTn (NCBI). The information of the sequences was later used to construct a phylogenetic tree (**Figure 1**).

## Discussion

The PIFs analyzed in the present study are commercialized throughout the Americas. Therefore, evaluating their microbiological quality allows determining aspects such as the hygienic conditions in which they were prepared, as well as identifying microbial hazards from probable recontamination occurring when they are supplemented with nutrients after pasteurization (Kent et al., 2015).

For APC, 72% of all analyzed powdered and liquid DP samples contained less than 3 log CFU. There were 11% of PM brands that contained between 4.0 and 4.7 log CFU/g originating only from the United States and Singapore; two products manufactured in Chile had values of 6.7 log CFU/g. Liquid DPs revealed five samples that ranged between 5.6 and 5.8 log CFU/g, four of which were produced in the United States and one in Chile. These APC values are within ranges reported by other authors (Iversen et al., 2004; Kim et al., 2011; Parra-Flores et al., 2015a). However, it was a concern to find counts greater than 5 log CFU/g in PM; these values were very high compared to results reported by Chap et al. (2009), who found 2% in this range. Heperkan et al. (2017) found values between 1.7 log for PIF and 4.9 log CFU/g for PM in a study of 80 PM samples; counts were similar to those determined in the present study. There is an evident need to control the contamination sources of DM products due to the wide range of microorganisms present in the APC and the higher susceptibility of infection in children of different ages who consume DPs (Chap et al., 2009).

Positivity was found for ENT in 55% of all analyzed samples, and there was a significant statistical relationship in the counts for commercial brand (*p* = 0.012) and manufacturer (*p* = 0.010). Eight PM and two liquid DPs obtained count means of 2 log CFU and 11 samples had values from 3.4 to 4.5 log CFU. Muytjens et al. (1988) encountered ENT in 52% of 141 evaluated formulas from 35 countries. On the other hand, ENT was found in 47% of PIF manufactured in Indonesia and Malaysia (Estuningsih et al., 2006), 22.5% in Ivory Coast (Yao et al., 2012), and 100% in Chile (Parra-Flores et al., 2015a). All these ENT counts are much higher than the values permitted according to the international standard of Codex Alimentarius Commission (2008), which requires the absence of this indicator in 10 g. In the present study, the high ENT positivity is compatible with the presence of several opportunistic microorganisms and pathogens associated with disease in infants reported in different publications (Food and Agriculture Organization and World Health Organization [FAO/WHO], 2006; Kent et al., 2015). Therefore, these findings should be analyzed in terms of risk associated with the consumption of PM by infants, the lack of control by manufacturers, and health authorities responsible for inspection in Chile. Although the association between the risk of falling ill with the consumption of ENT-contaminated PM has not yet been established with certainty, its absence in PM provides additional protection for newborns, especially the preterm, immunocompromised, and those with low (<2,500 g) and very low (<1,500 g) birth weight during the preparation, storage, and administration of infant feeding (Abdullah Sani et al., 2013).

Other microorganisms belonging to this family were also identified in the present study. *E. cloacae*, *K. pneumoniae*, and *E. hormaechei* were found. This situation does not seem altogether exceptional considering that other authors have also found these microorganisms of the ENT family in PIF (Iversen et al., 2004; Giammanco et al., 2011; Kim et al., 2011; Abdullah Sani et al., 2013), and especially for the risk associated with its ability to maintain itself for at least 8 months under desiccation conditions (Caubilla-Barron and Forsythe, 2007; Juma et al., 2016). Furthermore, the Food and Agriculture Organization and World Health Organization [FAO/WHO] (2004, 2006) also recognized that other ENT can be recovered from PIF and could present a risk to infants, although no reported cases had been confirmed at that time. Jackson et al. (2015) re-evaluated a reported *C. sakazakii* outbreak through the consumption of contaminated reconstituted PIF in Mexico. Using DNA sequencing, they demonstrated that the causative agents were misidentified strains of *E. hormaechei* and *Enterobacter* spp. Meanwhile, *E. hormaechei* has been shown to have clinical significance with the report of several outbreaks of sepsis in neonatal intensive care units in Brazil and the United States (Wenger et al., 1997; Campos et al., 2007; Townsend et al., 2008).

The high APC and ENT values can indicate a non-strict adherence to hygienic practices recommended for the preparation of PIF, which has been mentioned by other authors (Mullane et al., 2007; Jongenburger et al., 2011). This can imply a permanent risk for populations that usually consume this product.

The *C. sakazakii* incidence was 8.8% in the total of evaluated samples, particularly in 10 and 35% of samples produced in Singapore and Chile, respectively. This high positivity should give rise to greater control by the manufacturers and health authorities because of its high lethality, related neurological sequela, and risk of falling ill by *C. sakazakii* (Lai, 2001; Holý and Forsythe, 2014). An infection rate of 1 in 100,000 newborns has been estimated in the United States; this rate increases to 8.7 in 100,000 in infants weighing less than 1500 g, and 1 in 10,660 preterm infants with low birth weight (Hunter and Bean, 2013). In Holland, *Cronobacter* spp. causes from 0.5 to 0.7% of all the cases of meningitis in infants, with a probable range of infection of 0.00062 to 0.62 cases per year. When this probability is adjusted with all the cases that have occurred in the last 30 years, the projected probability is 0.53 cases of infection per year with a rate of 1 in 100,000 infants (Reij et al., 2009). There is no doubt that these values are greatly underestimated (Kucerova et al., 2011; Jason, 2015). Patrick et al. (2014) stated that the median age in adults is 59 for disease caused by *Cronobacter* spp., this value has been widely referred to by the lay press and the representatives of formula manufacturers. Parra-Flores et al. (2016) evaluated cell response variability of *C. sakazakii* after mild heat treatments using stochastic approaches and reported that these can better describe microbial single cell response than deterministic models. They found that the mean probability of illness from the initial inoculum size of 1 cell was less than 0.2 in all cases, while the mean probability of illness was greater than 0.7 in most cases for the inoculum size of 50 cells.

A principal aspect of our study was the correct identification of *Cronobacter* spp. and *C. sakazakii* species by several methods described in the literature (**Table 1**). When comparing the methods, a very good correlation was found for these methods by using different primers with *fusA* gene sequencing, which today enables the most accurate speciation because it follows the whole genome phylogeny and adjusts to taxonomic changes (Forsythe et al., 2014; Jackson et al., 2014; Xu et al., 2014; Alsonosi et al., 2015; Vojkovska et al., 2016). Therefore, the information generated when using molecular techniques can improve the confidence level in the identification and confirmation of presumptive strains even when molecular tests provide the best identification and phenotyping methods (Joseph et al., 2013; Jackson and Forsythe, 2016).

The robustness of the results was a primary facet in our decision to declare the national and international food alert and the massive recall of the products involved. There was another important point health authorities needed to consider, that is, the 2007 WHO recommendation to use water at >70°C to rehydrate PM to limit the risk of infection by *Cronobacter* spp. (World Health Organization [WHO], 2007). It was also recommended that rehydrated PM for children be administered within 2 h of its preparation or conservation under refrigeration at <4°C. This was the main idea of the publicity campaign launched by the authorities for the Chilean population.

Unfortunately, there are situations that warn us that microbiological control cannot be relaxed in food products consumed by hypersensitive populations, such as children and the elderly. For example, the case of the recall of *C. sakazakii*-contaminated PM destined for children in Argentina in 2015, and the recent contamination of Lactalis milk with *Salmonella* spp., which affected 83 European countries. However, recent studies demonstrate that the microbiological quality of these products is still inadequate even when we know that milk and DPs for child feeding are not sterile (Parra-Flores et al., 2015a).

In summary, an inadequate microbiological quality of powdered and liquid PM consumed by children under 10 was found in the present study. The presence of ENT and *C. sakazakii* was also identified, which is a wake-up call to manufacturers and public health regulatory authorities in Chile and throughout the Americas. It is therefore necessary to establish greater control of hygienic conditions in PM production and microbiological vigilance to prevent unnecessary risks for the child population that massively consumes these products (Koletzko et al., 2012). Disease caused in children as a consequence of any pathogen present in the PM they consume, requires manufacturers and health authorities to ensure the highest possible level of food safety (Food and Agriculture Organization and World Health Organization [FAO/WHO], 2006; Jason, 2015; Kent et al., 2015).

## Conclusion

Powdered infant milk formulas (PIF) are not sterile products; according to the specifications established by the Codex Alimentarius, this type of product should be treated as a possible food safety issue for high risk populations, such infants and neonates, due to the presence of the *C. sakazakii* pathogen. A total of 11% of the powdered milk brands contained APCs between 4.0 and 4.7 log CFU/g, which is considered as the rejection level by the updated Chilean Food Sanitary Regulations (RSA). Of all the samples, 55% contained *Enterobacteriaceae*; *E. cloacae*, *E. hormaechei*, and *K. pneumoniae* were identified. The overall incidence of *C. sakazakii* was 8.8%, which was found in samples produced either in Chile or Singapore.

Based on this information, the Chilean Ministry of Health decreed a national and international food alert and recalled all the product batches from supermarkets and pharmacies that tested positive in the study. After the first survey conducted for PIF contaminated with *Cronobacter* spp., it was pointed out that this microorganism was present and represented a risk that was not considered in the Chilean food safety standards. The RSA therefore included a new regulation for *Cronobacter* spp. in PIF in November 2017 because of social media pressure and the scientific results provided by our team.

## Author Contributions

JP-F conceived the experiments. JP-F, FC-L, and JA designed the experiments. NV-R, AC, and JP-F conducted the laboratory work. JP-F, NV-R, AR, and FC-L drafted the manuscript. All authors reviewed and approved the final manuscript.

## Conflict of Interest Statement

The authors declare that the research was conducted in the absence of any commercial or financial relationships that could be construed as a potential conflict of interest.
